# Porcine deltacoronavirus nsp5 antagonizes type I interferon signaling by cleaving IFIT3

**DOI:** 10.1128/jvi.01682-23

**Published:** 2024-01-30

**Authors:** Haixin Huang, Xiaoxiao Lei, Chenchen Zhao, Yan Qin, Yuying Li, Xinyu Zhang, Chengkai Li, Tian Lan, Baopeng Zhao, Wenchao Sun, Huijun Lu, Ningyi Jin

**Affiliations:** 1 College of Veterinary Medicine, Northwest A&F University, Xianyang, Shaanxi, China; 2 Institute of Virology, Wenzhou University, Wenzhou, Zhejiang, China; 3 Changchun Institute of Veterinary Medicine, Chinese Academy of Agricultural Sciences, Changchun, Jilin, China; Loyola University Chicago - Health Sciences Campus, Maywood, Illinois, USA

**Keywords:** PDCoV, nsp5, IFIT3, cleavage, type I interferon signaling

## Abstract

Porcine deltacoronavirus (PDCoV) has caused enormous economic losses to the global pig industry. However, the immune escape mechanism of PDCoV remains to be fully clarified. Transcriptomic analysis revealed a high abundance of interferon (IFN)-induced protein with tetratricopeptide repeats 3 (IFIT3) transcripts after PDCoV infection, which initially implied a correlation between IFIT3 and PDCoV. Further studies showed that PDCoV nsp5 could antagonize the host type I interferon signaling pathway by cleaving IFIT3. We demonstrated that PDCoV nsp5 cleaved porcine IFIT3 (pIFIT3) at Gln-406. Similar cleavage of endogenous IFIT3 has also been observed in PDCoV-infected cells. The pIFIT3-Q406A mutant was resistant to nsp5-mediated cleavage and exhibited a greater ability to inhibit PDCoV infection than wild-type pIFIT3. Furthermore, we found that cleavage of IFIT3 is a common characteristic of nsp5 proteins of human coronaviruses, albeit not alphacoronavirus. This finding suggests that the cleavage of IFIT3 is an important mechanism by which PDCoV nsp5 antagonizes IFN signaling. Our study provides new insights into the mechanisms by which PDCoV antagonizes the host innate immune response.

**IMPORTANCE:**

Porcine deltacoronavirus (PDCoV) is a potential emerging zoonotic pathogen, and studies on the prevalence and pathogenesis of PDCoV are ongoing. The main protease (nsp5) of PDCoV provides an excellent target for antivirals due to its essential and conserved function in the viral replication cycle. Previous studies have revealed that nsp5 of PDCoV antagonizes type I interferon (IFN) production by targeting the interferon-stimulated genes. Here, we provide the first demonstration that nsp5 of PDCoV antagonizes IFN signaling by cleaving IFIT3, which affects the IFN response after PDCoV infection. Our findings reveal that PDCoV nsp5 is an important interferon antagonist and enhance the understanding of immune evasion by deltacoronaviruses.

## INTRODUCTION

Porcine deltacoronavirus (PDCoV) is a newly emerging member of the *Deltacoronavirus* (δ-CoVs) genus (1). δ-CoVs are the last identified *Coronaviridae* subfamily genus and are reported to infect both birds and mammals (2). Recent studies have shown that PDCoV can also infect calves, chickens, and humans, raising the possibility of cross-species transmission (3, 4). PDCoV is an important intestinal and respiratory pathogen in pigs that can cause watery diarrhea, vomiting, dehydration, and high mortality in suckling piglets. In 2012, PDCoV was first detected in Hong Kong (1). From 2014 to 2020, PDCoV was widely reported in the United States (5), South Korea (6), Thailand (7), Canada (8), and Mainland China (9, 10), exciting a substantial financial toll on the pig industry. PDCoV is an enveloped, single-stranded, positive-sense RNA virus whose genome is approximately 25.4 kb in length and is arranged in the order of 5′ UTR-open reading frame 1a/1b-S-E-M-NS6-N-NS7-NS7a-3′ UTR. Papain-like protease (PL^pro^, nsp3) and the main protease, also known as three chymotrypsin-like protease (M^pro^, 3CL^pro^, or nsp5), are essential for viral replication and assembly, cleaving the viral genome to produce 15 mature nonstructural proteins (Nsp2-16) (11). Nsp5 of coronaviruses has a conserved three-domain structure and forms a unique chymotrypsin-like folding structure with a molecular weight of approximately 32 kDa (12–14). The function of nsp5 is not limited to the cleavage of only viral nonstructural proteins; nsp5 can also cleave host proteins, such as DCP1A, HDAC, STAT2, SQSTM1, MAGED2, and POLDIP3, an ability that is reflected in the mechanisms by which different coronaviruses evade host immunity (15–20). Nsp5 is an attractive drug target because it plays an important role in cleaving viral polyproteins into functional proteins as well as in cleaving host proteins for evasion of host innate immunity.

Interferons (IFNs) are antiviral cytokines secreted by host cells in response to various pathogens that trigger the transcription of IFN-stimulated genes (ISGs) to elicit protective immune defense responses (21). IFN-induced protein with tetratricopeptide repeats (IFITs) family members is encoded by some of the hundreds of ISGs. A variety of protein-protein interactions are mediated by IFIT1/ISG56, IFIT2/ISG54, IFIT3/ISG60, and IFIT5/ISG58, which play a role in translation initiation, double-stranded RNA signaling, and virus replication (22). IFITs play an important role in pathogen infection and have been reported to inhibit the replication of a variety of viruses, such as Seneca virus A, highly pathogenic porcine reproductive and respiratory syndrome virus (HP-PRRSV), and severe acute respiratory syndrome coronavirus 2 (23–25). In addition, herpes simplex virus 1 (HSV-1) tegument protein UL41 was found to diminish the accumulation of IFIT3 mRNA to abrogate its antiviral activity (26). However, little is known about the function of IFIT3 upon PDCoV infection.

Sendai virus (SeV) is a RIG-I-mediated recognition RNA virus that can activate type I interferon signaling pathway (27). Previous study reported that accessory protein NS6 of PDCoV significantly inhibits SeV-induced interferon beta (IFN-β) production as well as the activation of transcription factors IRF3 and NF-κB (28). In our study, we confirmed that PDCoV strongly inhibited the SeV-induced ISG and cytokine production in ST cells. IFIT3, an important IFN-stimulated gene, has been studied extensively in the context of RNA and DNA viruses, but its effect on coronaviruses is still unclear. For the first time, we demonstrated that PDCoV nsp5 antagonizes type I IFN signaling by targeting IFIT3. In addition, we revealed that nsp5 proteins of other coronaviruses, such as Middle East respiratory syndrome coronavirus (MERS-CoV), SARS-CoV and SARS-CoV-2, also possess protease activity to cleave human IFIT3. This study helps to close the research gap in functional analysis of the PDCoV nsp5 protein.

## RESULTS

### IFIT3 is induced by PDCoV

**Fig 1:**
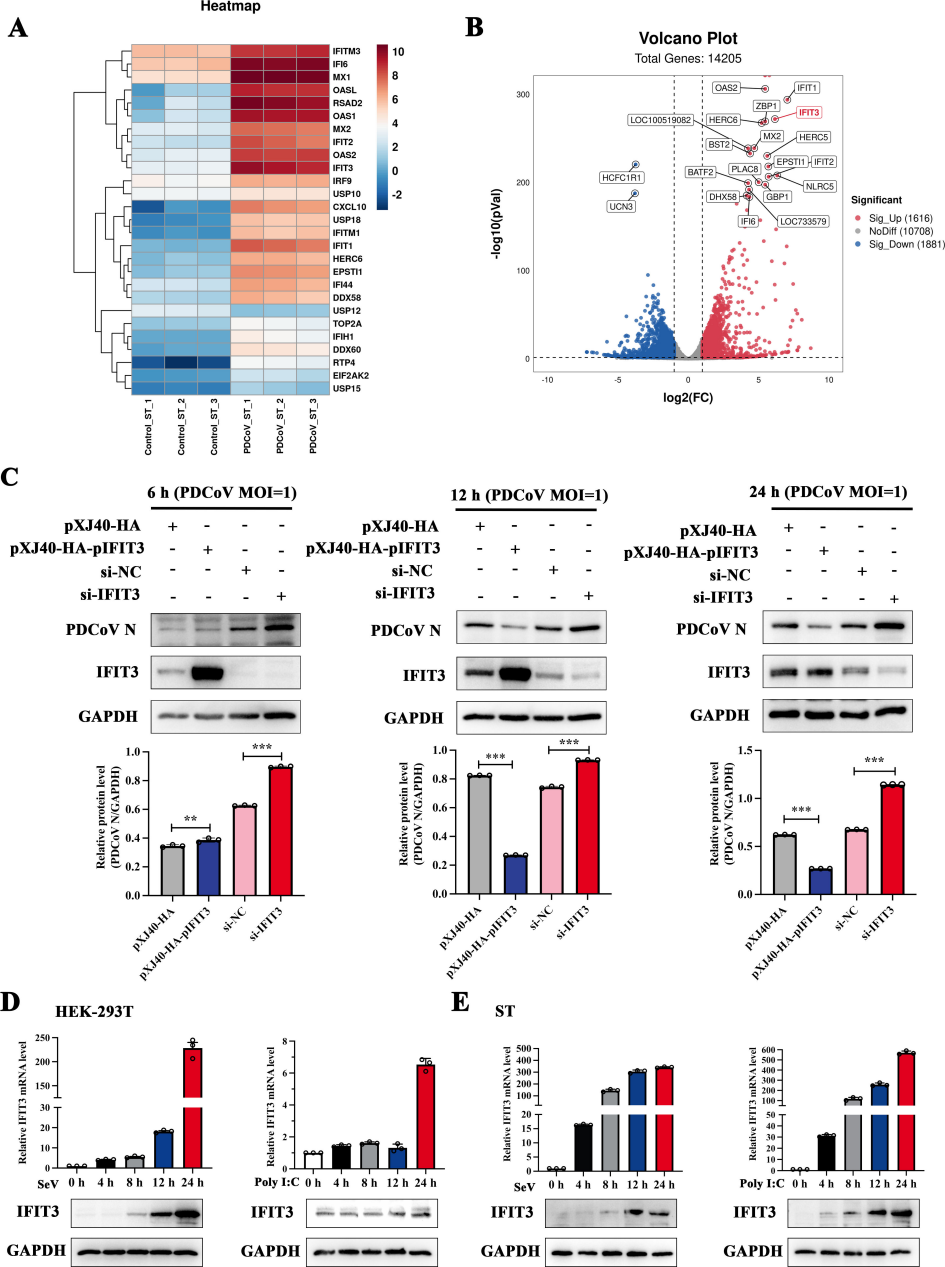
PDCoV and poly(I:C) induced IFIT3 expression. (**A**) Heatmap showing the fold changes in the expression of genes in PDCoV-infected ST cells compared with mock-infected ST cells at 24 h. (**B**) Volcano plot showing the global differentially expressed genes in PDCoV-infected ST cells compared with mock-infected ST cells at 24 h. (**C**) pXJ40-HA-vector, pXJ40-HA-pIFIT3, IFIT3 siRNA, and negative control siRNA were transfected into ST cells. After 24 h of transfection, the cells were infected with PDCoV (MOI = 1) for 6, 12, and 24 h and were then harvested for Western blot analysis. Relative expression levels in the pXJ40-HA-vector, pXJ40-HA-pIFIT3, IFIT3 siRNA, and negative control siRNA groups were determined and normalized to glyceraldehyde-3-phosphate dehydrogenase (GAPDH) expression using ImageJ. (**D and E**) HEK-293T and ST cells were treated with poly(I:C) (2 µg/mL) for 0, 4, 8, 12, or 24 h. And HEK-293T and ST cells were infected with SeV for 0, 4, 8, 12, or 24 h. The cells were lysed and collected, and the IFIT3 mRNA levels were then measured by RT-qPCR. The protein expression levels of IFIT3 and GAPDH were measured by Western blotting. All data are reported as means ± SDs. For all experiments, **P < 0.01, and ***P < 0.001 were considered to indicate statistical significance.

To study host gene interactions during PDCoV infection, we infected ST cells with PDCoV, and after 24 hpi, when most cells were infected, we performed RNA-seq analysis. IFIT3, which is essential during viral infection, was upregulated by PDCoV infection (Fig. 1A and B). To further confirm the effect of IFIT3 on PDCoV replication, three IFIT3-specific siRNAs and one negative control siRNA (NC-siRNA) were designed and synthesized to knock down IFIT3 expression (Fig. S1A and B). Next, we transfected ST cells with porcine IFIT3 (hereafter abbreviated pIFIT3) expression plasmid and performed an indirect immunofluorescence assay. The pXJ40-HA-pIFIT3 group contained fewer PDCoV virions than the empty vector group. In contrast, compared with NC-siRNA-transfected cells, IFIT3 siRNA-transfected cells contained a greater abundance of PDCoV protein, as determined by the immune-fluorescence assays (Fig. S1C and D). Next, we transfected NC-siRNA, IFIT3 siRNA, empty vector, and pXJ40-HA-pIFIT3 into ST cells and then infected the cells with PDCoV (MOI = 1) for 6, 12, and 24 h. As shown in Fig. 1C, during PDCoV infection, IFIT3 overexpression reduced PDCoV N protein expression in the ST cells, and conversely, reducing the expression of IFIT3 promoted PDCoV N protein expression. These results suggested that IFIT3 can significantly inhibit PDCoV replication. Western blot and quantitative real-time PCR (RT**-**qPCR) analyses were performed to detect the induction of IFIT3 upon SeV and poly(I:C) stimulation at 0, 4, 8, 12, and 24 h. IFIT3 mRNA and protein expression were markedly induced at different time points after stimulation in HEK-293T and ST cells (Fig. 1D and E).

### IFIT3 positively regulates the type I IFN signaling pathway

**Fig 2:**
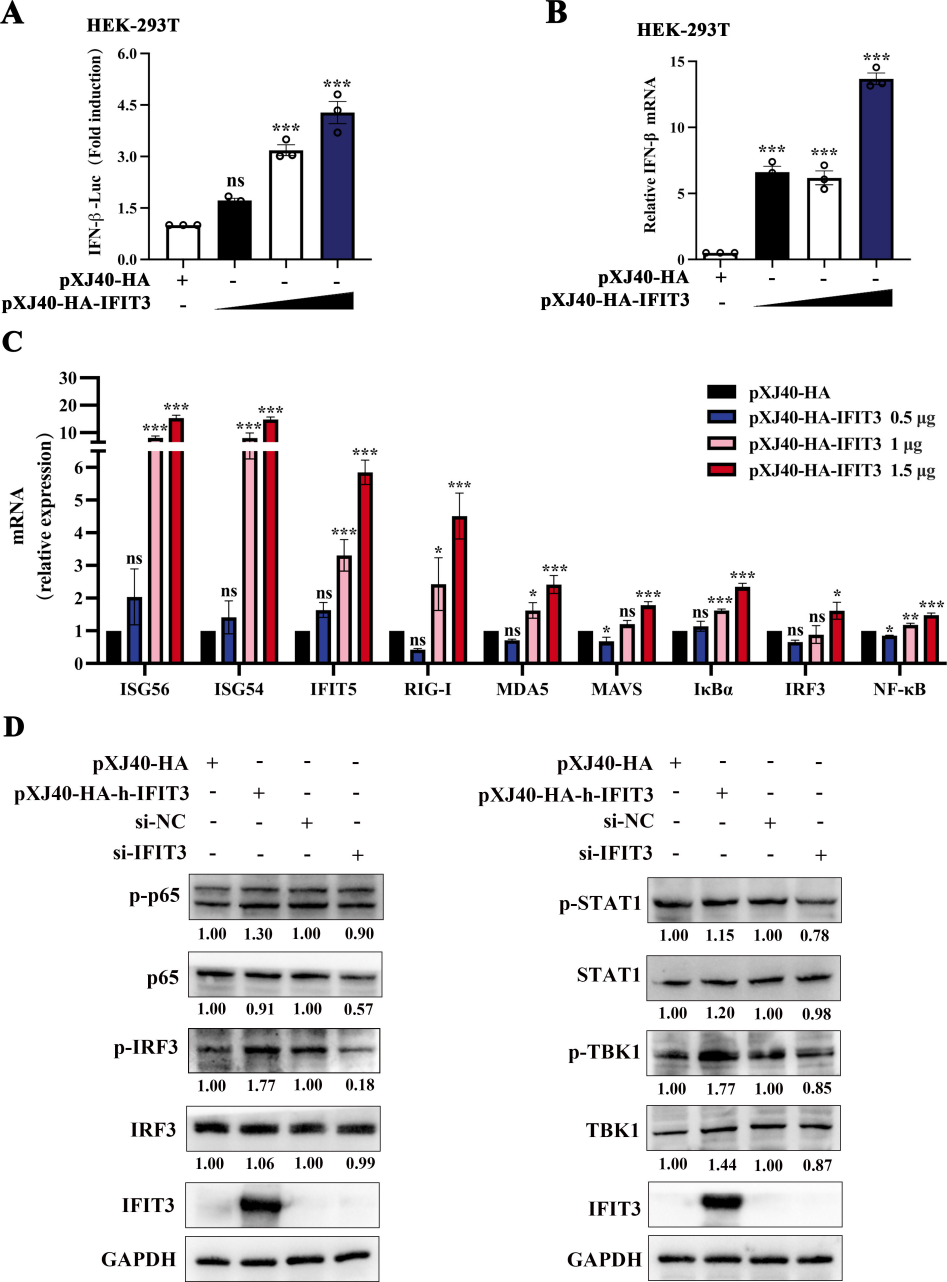
IFIT3 activates the type I IFN signaling pathway. (**A**) HEK-293T cells were cotransfected with the IFN-β Luc and pRL-TK plasmids, along with 0.5, 1, or 1.5 µg of the pXJ40-HA-pIFIT3 expression plasmid for 30 h. The cells were lysed and subjected to a dual-luciferase assay. (**B**) HEK-293T cells were transfected with 0.5, 1, or 1.5 µg of the pXJ40-HA-pIFIT3 for 30 h. The cells were collected, and the IFN-β mRNA level was measured by RT-qPCR. (**C**) HEK-293T cells were transfected with 0.5, 1, or 1.5 µg of the pXJ40-HA-pIFIT3. After 30 h, the cells were collected, and the ISG56, ISG54, IFIT5, RIG-I, MDA-5, MAVs, IκBα , IRF3, and NF-κB mRNA levels were measured by RT-qPCR. (**D**) pXJ40-HA-vector, pXJ40-HA-pIFIT3, IFIT3 siRNA, and negative control siRNA were transfected into HEK-293T cells. After 30 h of transfection, the cells were collected and analyzed by Western blotting with anti-P65, anti-phospho-P65, anti-IRF3, anti-phospho-IRF3, anti-STAT1, anti-phospho-STAT1, anti-TBK1, anti-phospho-TBK1, anti-IFIT3, and anti-glyceraldehyde-3-phosphate dehydrogenase (GAPDH) antibodies. Relative levels of P65, phospho-P65, IRF3, phospho-IRF3, STAT1, phospho-STAT1, TBK1, and phospho-TBK1 were quantified and normalized to the GAPDH expression level using ImageJ. All data are reported as the means ± SDs. For all experiments, *P < 0.05, **P < 0.01, and ***P < 0.001 were considered to indicate statistical significance. ns, nonsignificant difference.

To further explore the mechanism by which IFIT3 inhibits viral replication, we performed a dose-response assay to evaluate IFIT3-induced IFN-β promoter activity in HEK-293T cells. The results suggested that IFIT3 can increase IFN-β promoter activity and the IFN-β mRNA expression level (Fig. 2A and B). Then, we measured the mRNA levels of ISG56, ISG54, IFIT5, RIG-I, MDA-5, MAVs, IκBα, IRF3, and NF-κB after transfection of HEK-293T cells with different amounts of IFIT3. As shown in Fig. 2C, IFIT3 was identified as a significantly upregulated ISG and cytokine by RT**-**qPCR analysis. Moreover, in HEK-293T cells, overexpression of IFIT3 increased the phosphorylation of STAT1, TBK1, IRF3, and p65, while knockdown of IFIT3 inhibited their activation (Fig. 2D). Thus, IFIT3 can directly activate the type I IFN signaling pathway.

### PDCoV Nsp5 inhibits IFN-β and ISG production

**Fig 3:**
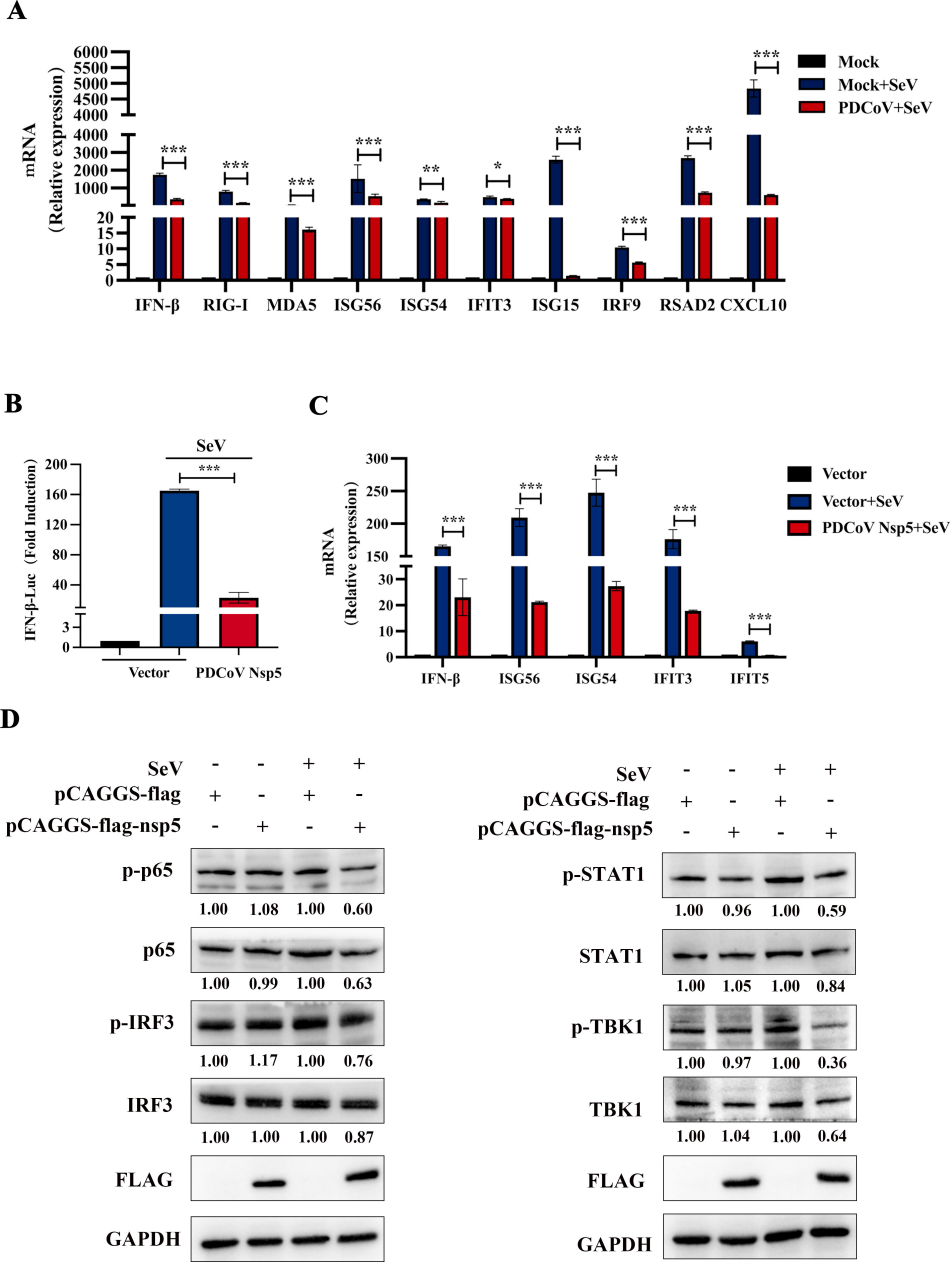
PDCoV nsp5 inhibits the SeV-induced type I IFN signaling pathway. (**A**) ST cells were infected with the PDCoV (MOI = 1). After 12 h, the cells were further infected or mock infected with SeV or mock infected for 12 h. Cells were collected at 12 h post infection, and the IFN-β, RIG-I, MDA-5, ISG56, ISG54, IFIT3, ISG15, IRF9, RSAD2, and CXCL10 mRNA levels were measured by RT-qPCR. (**B**) HEK-293T cells were cotransfected with the IFN-β Luc and pRL-TK plasmids, along with 1.2 µg of pCAGGS-flag-nsp5 or an empty vector. After 24 h, the cells were further infected or mock infected with SeV or mock infected for 12 h. The cells were lysed and subjected to a dual-luciferase assay. (**C**) ST cells were transfected with 1.2 µg of pCAGGS-flag-nsp5 or an empty vector. After 24 h of initial transfection, the cells were further infected or mock infected with SeV or mock infected. The cells and supernatants were collected at 12 h post infection, and the IFN-β, ISG56, ISG54, IFIT3, and IFIT5 mRNA levels were measured by RT-qPCR. (**D**) HEK-293T cells were transfected with the pCAGGS-flag-nsp5 for 24 h and stimulated with SeV for 12 h. Cell lysates were analyzed by Western blotting with anti-P65, anti-phospho-P65, anti-IRF3, anti-phospho-IRF3, anti-STAT1, anti-phospho-STAT1, anti-TBK1, anti-phospho-TBK1, anti-IFIT3, and anti-glyceraldehyde-3-phosphate dehydrogenase (GAPDH) antibodies. Relative levels of P65, phospho-P65, IRF3, phospho-IRF3, STAT1, phospho-STAT1, TBK1, and phospho-TBK1 were quantified and normalized to the GAPDH expression level using ImageJ. All data are reported as the means ± SDs. For all experiments, *P < 0.05, **P < 0.01, and ***P < 0.001 were considered to indicate statistical significance.

We next examined the effect of PDCoV infection on the innate immune response to SeV, which activates the type I IFN signaling pathway (29, 30). However, PDCoV strongly inhibited the SeV-induced ISG and cytokine production in ST cells (Fig. 3A). HEK-293T cells were transfected with empty vector or pCAGGS-flag-nsp5 along with a luciferase reporter plasmid containing the IFN-β promoter (IFN-β-Luc) and a control pRL-TK plasmid. After 24 h, the cells were infected with SeV, mimicking RLR activation by PDCoV. The activity of the IFN-β promotor was strongly impaired by nsp5 (Fig. 3B). And PDCoV nsp5 also inhibited SeV-induced IFN-β and ISG mRNA expression (Fig. 3C). To verify the antagonistic effect of PDCoV nsp5 on IFN production, the N-terminus of RIG-I, a well-known upstream activator of canonical IFN signaling, was used as a potent inducer of IFN production (31). Expression of the PDCoV nsp5 protein inhibited RIG-I-induced activation of IFN-β and ISG production (Fig. S2A). After transfection of pCAGGS-flag-nsp5 or empty vector for 24 h, HEK-293T cells were then stimulated with SeV for 12 h. Western blot analysis showed that PDCoV nsp5 inhibited SeV-induced STAT1, TBK1, IRF3, and p65 phosphorylation (Fig. 3D). Collectively, our data reveal that PDCoV nsp5 inhibits the type I IFN signaling pathway.

### PDCoV Nsp5 targets IFIT3 for cleavage

**Fig 4:**
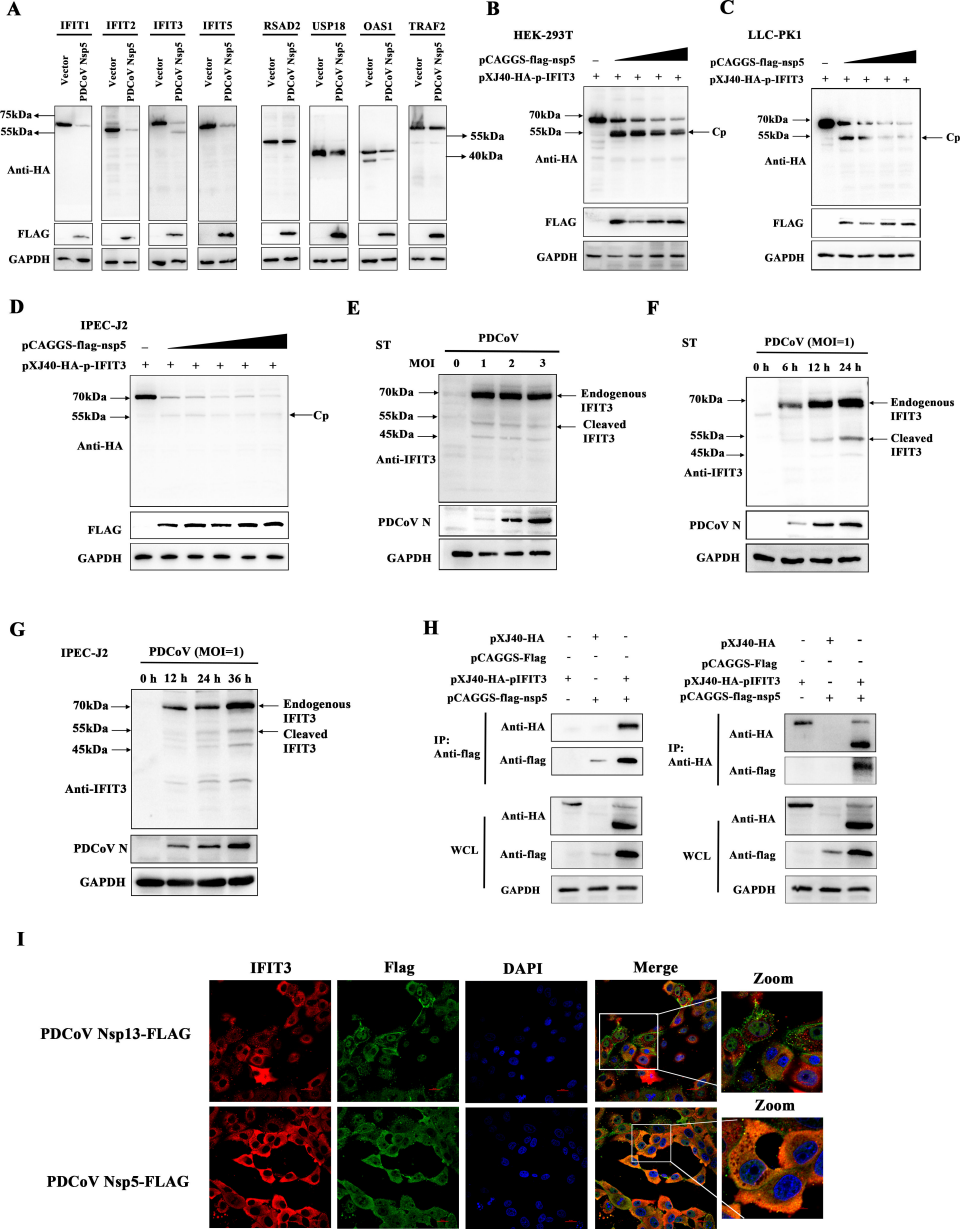
PDCoV nsp5 targets IFIT3 for cleavage. (**A**) HEK-293T cells were cultured in 6-well plates and cotransfected with the PDCoV nsp5 expression plasmid or empty vector along with 1.2µg of the HA-tagged IFIT1, IFIT2, IFIT3, IFIT5, RSAD2, USP18, OAS1, or TRAF2 expression plasmid. After 30h, the cells were lysed and analyzed by Western blotting with an anti-HA antibody. (**B–D**) HEK-293T, LLC-PK1, and IPEC-J2 cells were cotransfected with pXJ40-HA-pIFIT3 and various amounts of pCAGGS-flag-nsp5. After 30 h, the cells were lysed for Western blotting. The cleavage products are labeled “Cp.” (**E**) ST cells were transfected with 1.2 µg/well pXJ40-HA-pIFIT3. After 24 h of transfection, the ST cells were infected with PDCoV at MOIs of 1, 2, and 3. After 24 h, the cells were lysed for Western blotting. (**F**) ST cells were infected with PDCoV (MOI = 1), and the cells and supernatants were collected at 0, 6, 12, and 24 h for analysis by Western blot analysis. (**G**) IPEC-J2 cells were infected with PDCoV (MOI = 1), and the cells and supernatants were collected at 0, 12, 24, and 36 h for analysis by Western blot analysis. (**H**) HEK-293T cells were transfected with expression constructs encoding pCAGGS-flag-nsp5 and pXJ40-HA-pIFIT3. The cells were lysed 30 h after transfection and subjected to immunoprecipitation with an anti-FLAG antibody or anti-HA antibody. The whole-cell lysates (WCLs) and immunoprecipitation (IP) complexes were analyzed by immunoblotting using anti-FLAG, anti-HA, or anti-glyceraldehyde-3-phosphate dehydrogenase (GAPDH) antibodies. (**I**) ST cells were transfected with PDCoV nsp5 and nsp13. After 24 h, the cells were fixed and then stained with a rabbit monoclonal antibody against IFIT3 and a mouse anti-flag tag antibody prior to incubation with an Alexa Fluor 488-conjugated goat anti-mouse IgG antibody (green) or Alexa Fluor 594-conjugated goat anti-rabbit IgG antibody (red). Nuclei were stained with DAPI (blue).

To further analyze the molecular mechanisms by which IFIT3 and ISGs are regulated by PDCoV nsp5,pCAGGS-flag-nsp5 was cotransfected with the IFIT1, IFIT2, IFIT3, IFIT5, RSAD2, USP18, OAS1, and TRAF2 expression plasmids into HEK-293T cells. The expression levels of IFIT1, IFIT2, IFIT3, IFIT5, RSAD2, USP18, and OAS1 were reduced when PDCoV nsp5 was expressed. Interestingly, a band associated with a faster-migrating protein was observed in cells cotransfected with pXJ40-HA-pIFIT3 and pCAGGS-flag-nsp5, indicating that PDCoV nsp5 mediates the cleavage of pIFIT3 (Fig. 4A). To further confirm that pIFIT3 is a possible target cleaved by PDCoV nsp5, pXJ40-HA-pIFIT3 was cotransfected with different doses of pCAGGS-flag-nsp5 into HEK-293T, LLC-PK1, and IPEC-J2 cells. Western blot analysis showed that the expression levels of pIFIT3 and the cleavage product (hereafter abbreviated Cp) gradually decreased with the increasing doses of PDCoV nsp5 (Fig. 4B through D). Additionally, to identify the effect of PDCoV-infected systems on the expression of pIFIT3, we examined ST cells infected with different MOIs of PDCoV and detected cleavage products of pIFIT3 in cells infected with PDCoV (Fig. 4E). As shown in Fig. 4F and G, the cleavage products of pIFIT3 were also detected in ST cells and IPEC-J2 cells at different time points after PDCoV infection. Next, HEK-293T cells were cotransfected with pCAGGS-flag-nsp5 and pXJ40-HA-pIFIT3, and coimmunoprecipitation (Co-IP) was used to detect the interaction. pIFIT3 efficiently coimmunoprecipitated with the nsp5 protein (Fig. 4H). Based on these results, pIFIT3 directly binds to nsp5 of PDCoV. In addition, we performed confocal microscopy to determine whether pIFIT3 can colocalize with nsp5 of PDCoV and found cytoplasmic colocalization of pIFIT3 with the nsp5 protein but not the nsp13 protein of PDCoV (Fig. 4I). These results demonstrated the specificity of the interaction between PDCoV nsp5 and pIFIT3.

### PDCoV Nsp5-mediated inhibition of IFIT3 depends on its protease activity

**Fig 5:**
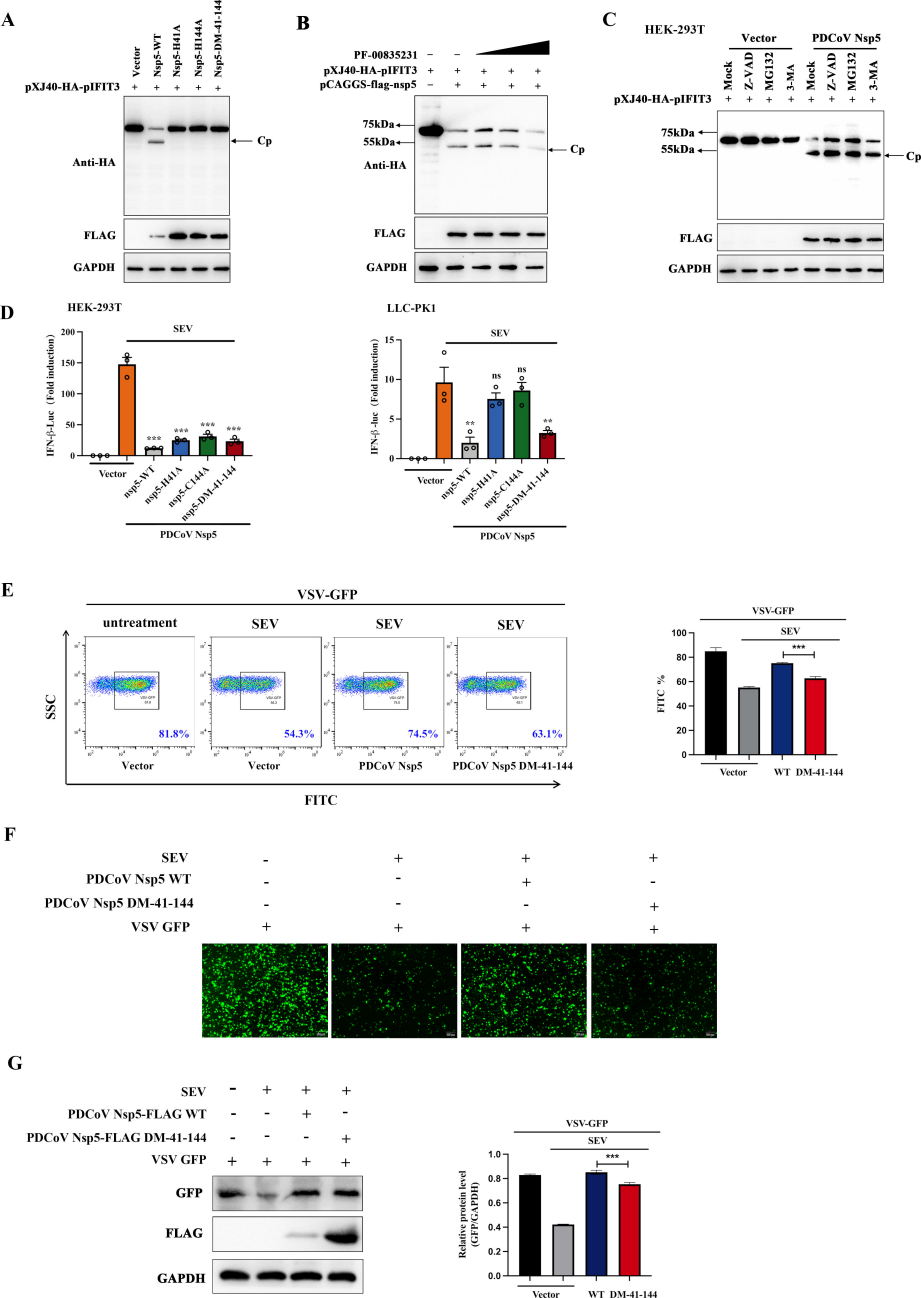
PDCoV nsp5 cleaves IFIT3 via its protease activity. (**A**) HEK-293T cells were cotransfected with plasmids expressing the wild-type PDCoV nsp5 or one of protease-defective mutants (C144A and H41A) and the pXJ40-HA-pIFIT3. After 30 h, the cells were lysed, and supernatants were used for Western blotting. (**B**) HEK-293T cells were cotransfected with pXJ40-HA-pIFIT3 and pCAGGS-flag-nsp5. After 24 h, the cells were mock treated or treated with PF-00835231 (10 µM). After 12 h, the cells were lysed for Western blotting. (**C**) HEK-293T cells were cotransfected with plasmids expressing the wild-type PDCoV nsp5 and pXJ40-HA-pIFIT3. After 24 h, the cells were treated with MG132 (10 µM), Z-VAD-FMK (25 µM), or 3-MA (10 µM) for 12 h. Cell lysates were prepared and analyzed by Western blotting. (**D**) HEK-293T and LLC-PK1 cells were cotransfected with the IFN-β-Luc and pRL-TK plasmids, along with 1.2 µg of pCAGGS-flag-nsp5, one of its protease-defective mutants (C144A, H41A, or DM-41-144) , or empty vector. After 30 h, the cells were lysed and subjected to a dual-luciferase assay. (**E–G**) HEK-293T cells seeded in a 12-well plate were transfected with equal amounts of plasmids encoding PDCoV nsp5 or one of its protease-defective mutants (C144A, H41A, and DM-41-144) for 24 h and were then infected with SeV for 12 h. The cell supernatants were then collected, treated with UV irradiation for 30 min, and added to a new 12-well plate of HEK-293T cells for 24 h. The IFN-treated cells were then inoculated with VSV-GFP for 12 h, and GFP expression was detected by flow cytometry, immunofluorescence staining, and Western blotting. Relative expression levels of GFP were quantified and normalized to the glyceraldehyde-3-phosphate dehydrogenase (GAPDH) expression level using ImageJ. All data are reported as the means ± SDs. For all experiments, **P < 0.01, and ***P < 0.001 were considered to indicate statistical significance. ns, nonsignificant difference.

It has been reported that H41 and C144 of PDCoV nsp5 are critical for its protease activity (19). In our study, we investigated whether PDCoV nsp5 cleaves pIFIT3 via its protease activity. We constructed plasmids expressing three mutants of nsp5 (H41A, C144A, and DM-H41A-C144A) and cotransfected them with pXJ40-HA-pIFIT3 into HEK-293T cells. The results suggested that wild-type nsp5 cleaved pIFIT3 successfully, while none of the three mutants did not (Fig. 5A). Chymotrypsin-related 3C-like protease (3CL^pro^) is encoded by nsp5. PF-00835231 is an inhibitor of the main viral protease 3CL^pro^ (32). As shown in Fig. 5B, the level of Cp decreased with increasing PF-00835231 treatment. Then, PDCoV nsp5 or empty vector was cotransfected with pXJ40-HA-pIFIT3, and the transfected cells were then treated with the proteasome inhibitor MG132, the caspase inhibitor Z-VAD-FMK, or the autophagy inhibitor 3-methyladenine (3-MA). Compared with that in the corresponding empty vector groups, the cleavage activity of PDCoV nsp5 was not affected by the inhibitors (Fig. 5C). The antiviral activities of wild-type PDCoV nsp5 and its mutants were then examined in HEK-293T cells and LLC-PK1 cells. As shown in Fig. 5D, wild--type PDCoV nsp5 exhibited the most significant inhibitory effects on SeV-stimulated IFN-β promoter activity among the mutants, as determined by a dual-luciferase reporter assay. Furthermore, the endogenous IFN expression level in HEK-293T cells transfected with plasmids encoding PDCoV nsp5 and its mutants (DM-H41A-C144A) was evaluated by an IFN bioassay using IFN-sensitive vesicular stomatitis virus-green fluorescent protein (VSV-GFP). SeV-stimulated HEK-293T cells were used as a positive control. The GFP-positive cells were then analyzed by flow cytometry. As shown in Fig. 5E, the percentage of GFP-positive cells among cells treated with culture supernatants from wild-type PDCoV nsp5-transfected cells was higher than that among cells treated with culture supernatants from PDCoV nsp5-DM-H41A-C144A plasmid-transfected cells, indicating that wild-type PDCoV nsp5 exhibited a stronger inhibitory effect on endogenous IFN production than PDCoV nsp5-DM-H41A-C144A. The IFN bioassay was also performed and analyzed by fluorescence microscopy and Western blotting. Consistent with the flow cytometry data, PDCoV nsp5 more strongly reversed the SeV-induced restriction of VSV-GFP replication than did PDCoV nsp5-DM-H41A-C144A (Fig. 5F and G). These findings indicated that PDCoV nsp5 markedly inhibits SeV-induced IFN production.

### PDCoV Nsp5 cleaves IFIT3 at Gln-406

**Fig 6:**
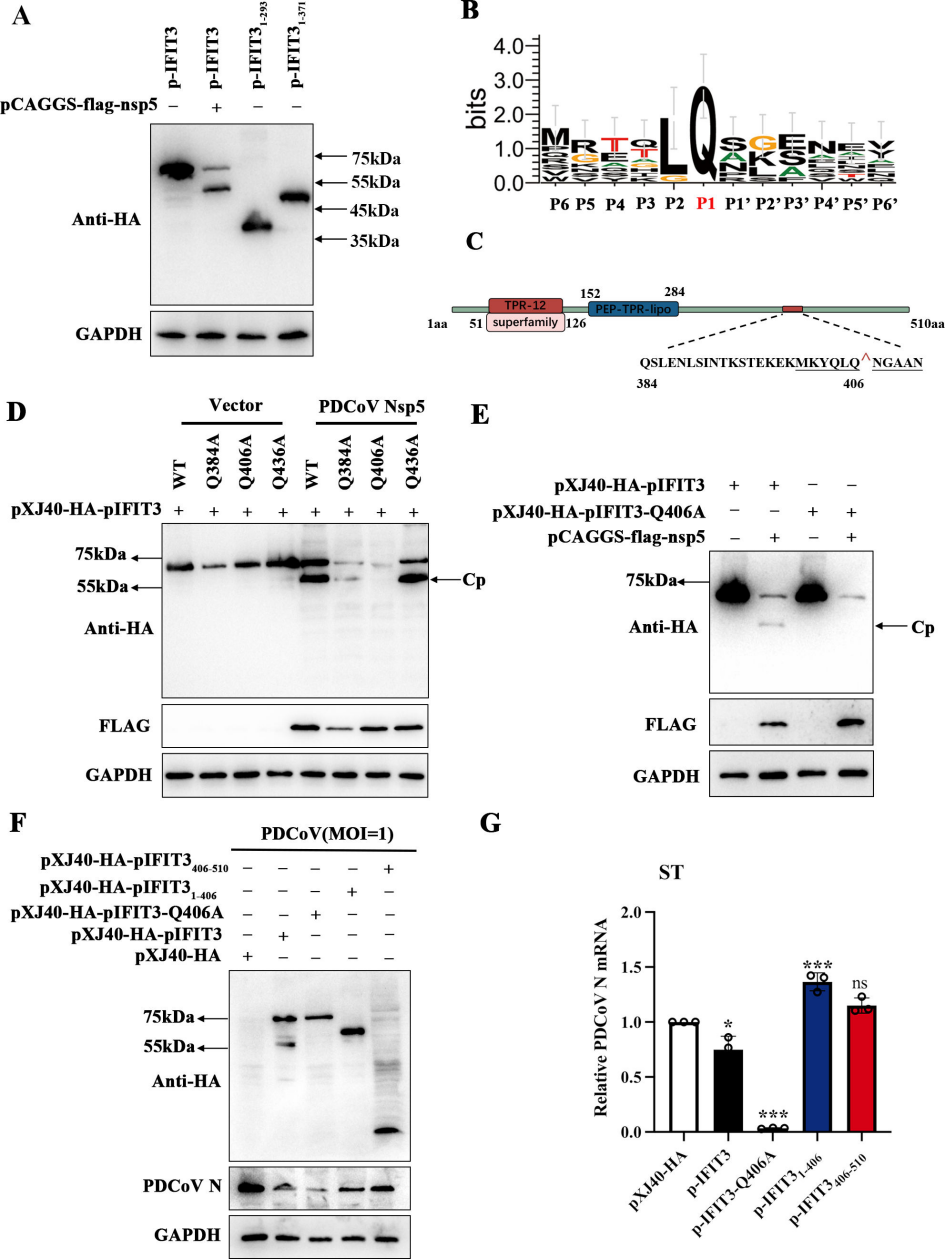
PDCoV nsp5 recognizes and cleaves IFIT3 at residue Q406. (**A**) HEK-293T cells were transfected with expression constructs encoding pXJ40-HA-pIFIT3, the pIFIT3_1-293_ truncated mutant, and pIFIT3_1-371_ truncated mutant and were collected after 30 h for Western blotting. (**B**) Sequence logo of the polyprotein junctions that are cleaved by PDCoV nsp5. An amino acid sequence logo of the substrate was generated by WebLogo version 3. (**C**) Schematic representation of pIFIT3 and its mutation sites. (**D**) HEK-293T cells were cotransfected with the pCAGGS-flag-nsp5 along with expression constructs encoding wild-type pIFIT3 or pIFIT3 mutants (pIFIT3-Q384A, pIFIT3-Q406A, and pIFIT3-Q436A). The cells were then lysed after 30 h and analyzed by Western blotting. (**E**) HEK-293T cells were co-transfected with the PDCoV nsp5 expression plasmid along with wild-type IFIT3 or pIFIT3-Q406A and collected after 30 h for Western blotting. (**F and G**) ST cells were transfected with empty vector, pIFIT3, pIFIT3-Q406A, pIFIT3_1-406_, or pIFIT3_406-510_. After 24 h, the cells were infected with PDCoV (MOI = 1) for 24 h and were then lysed and collected for analysis by Western blotting and RT-qPCR. All data are reported as the means ± SDs. For all experiments, *P < 0.05 and ***P < 0.001 were considered to indicate statistical significance. ns, nonsignificant difference.

Nsp5 cleaves polyproteins immediately downstream of a glutamine residue, as do the vast majority of the main proteases of coronaviruses (33–35). To investigate the site in pIFIT3 that is recognized by PDCoV nsp5, we constructed two pIFIT3 truncation mutants, pIFIT3_1-293_ and pIFIT3_1-371_, and cotransfected pIFIT3 along with PDCoV nsp5. We found that the molecular weight of the pIFIT3 cleavage product was approximately 55 kDa, and we predicted that the cleavage site may be near amino acid position 384 in the N-terminus (Fig. 6A). We used WebLogo, version 3 to generate the amino acid sequence logo of the substrate and noted that the Q residue at the P1 position is a common recognition site of coronavirus proteases. Therefore, we constructed pIFIT3 mutants with substitutions of the three Q residues between amino acids 384 and 436, namely, pIFIT3-Q384A, pIFIT3-Q406A, and pIFIT3-Q436A (Fig. 6B and C). Next, PDCoV nsp5 was cotransfected with these pIFIT3 mutants. Compared with that of the wild-type pIFIT3, pIFIT3-Q406A was resistant to nsp5-mediated cleavage, indicating a stronger antiviral function (Fig. 6D and E). To further verify the antiviral effects of different pIFIT3 truncations and identify the crucial mutation sites, we transfected pIFIT3_1-406_, pIFIT3_406-510_, and pIFIT3-Q406A into ST cells for 24 h, after which the cells were infected with PDCoV for measurement of the PDCoV N protein and mRNA expression levels. The results suggested that pIFIT3-Q406A significantly inhibited viral replication (Fig. 6F and G). These results reveal that the glutamine residue at position 406 of pIFIT3 is an important cleavage site for resistance to viral replication.

### The efficiency of Nsp5-mediated cleavage of IFIT3 differs across coronaviruses

**Fig 7:**
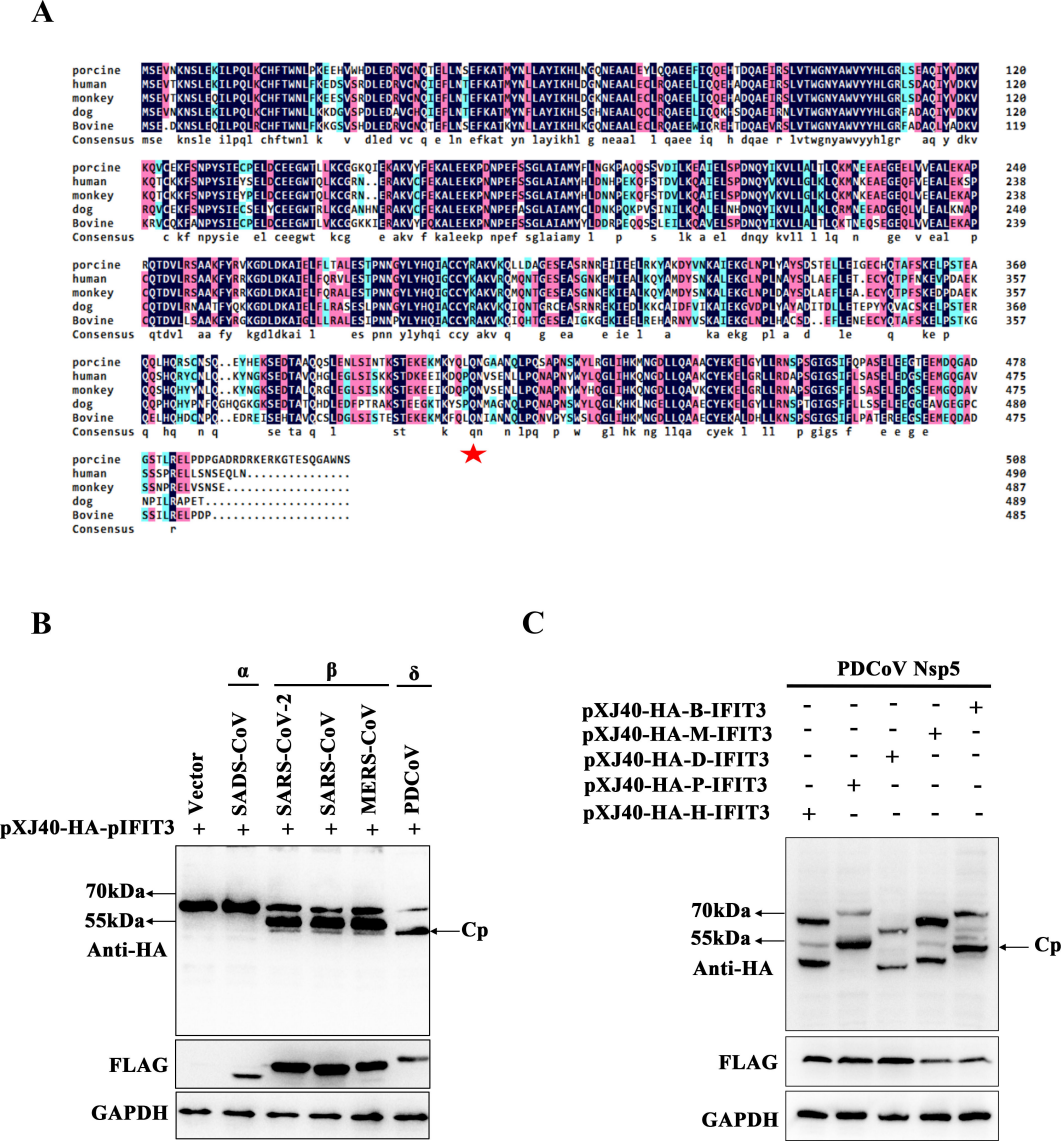
The efficiency of Nsp5-mediated cleavage of IFIT3 differs across coronaviruses. (**A**) Porcine, human, monkey, canine. and bovine sequences of IFIT3 were aligned with DNAMAN8. (**B**) HEK-293T cells were cultured in 12-well plates and cotransfected with pXJ40-HA-pIFIT3 and a plasmid encoding the nsp5 of SADS-CoV, SARS-CoV-2, SARS-CoV, MERS-CoV, or PDCoV. After 30 h, the cells were lysed and analyzed by Western blotting. (**C**) HEK-293T cells were cotransfected with PDCoV nsp5 and pXJ40-HA-B-IFIT3 (bovine), pXJ40-HA-M-IFIT3 (monkey), pXJ40-HA-D-IFIT3 (canine), pXJ40-HA-P-IFIT3 (porcine), and pXJ40-HA-H-IFIT3 (human). After 30 h, the cells were lysed and analyzed by Western blotting.

To verify whether nsp5 proteins of different coronavirus have different cleavage effects on host IFIT3, we analyzed the diversity of IFIT3 homologs across mammals. Multiple sequence alignment showed that the glutamine at position 406 of IFIT3 is conserved across the human, monkey, canine, bovine, and consensus IFIT3 sequences (Fig. 7A). As shown in Fig. 7B, we constructed the nsp5 proteins representing coronaviruses of three different coronavirus genera. Interestingly, the nsp5 protein of the alphacoronavirus swine acute diarrhoea syndrome coronavirus (SADS-CoV) did not cleave host IFIT3. In contrast, the nsp5 proteins of the beta-coronavirus SARS-CoV, SARS-CoV-2, and MERS-CoV exhibited cleavage functions consistent with that of PDCoV nsp5. It has been reported that PDCoV can infect multiple species. Therefore, we verified the cleavage effect of the PDCoV nsp5 protein on IFIT3 in bovines, monkeys, dogs, and humans. As expected, the PDCoV nsp5 protein cleaved IFIT3 proteins of multiple species (Fig. 7C). These results suggest that the cleavage effect of PDCoV nsp5 on IFIT3 is a main means by which viruses resist host innate immunity.

## DISCUSSION

Previous studies have demonstrated that IFIT3 is a host-intrinsic antiviral factor that restricts the replication of viruses such as HSV-1, dengue virus, and PRRSV (26, 36, 37). A previous study suggested that the human IFIT3 protein induces IFN signaling and inhibits adenovirus (Ad) immediate early gene expression (38). In addition to its direct involvement in the antiviral mechanism of translation inhibition, IFIT3 also acts as a molecular bridge between MAVS and TBK1, participating in the RIG-I signaling pathway and playing an indirect antiviral role (39). A recent study reported that transmissible gastroenteritis virus and PDCoV induced higher levels of IFIT3 than porcine epidemic diarrhea virus (PEDV) by the parallel comparison of transcriptomics data sets analysis, indicating that IFIT3 plays a crucial role in the IFN responses (40). PEDV nsp16 has been demonstrated to reduce IFIT1, IFIT2, and IFIT3 mRNA levels to hijack IFN signaling (41). Indeed, a recombinant rabies virus (RABV) expressing IFIT3 displayed a lower pathogenicity than the parental RABV in C57BL/6 mice, and IFIT3-deficient mice exhibited higher susceptibility to RABV infection and higher mortality during RABV infection; in addition, coexpression of IFIT2 and IFIT3 could more effectively inhibited RABV replication *in vitro* (42). However, the relative impact of IFIT3 in cells infected with PDCoV is unclear.

**Fig 8:**
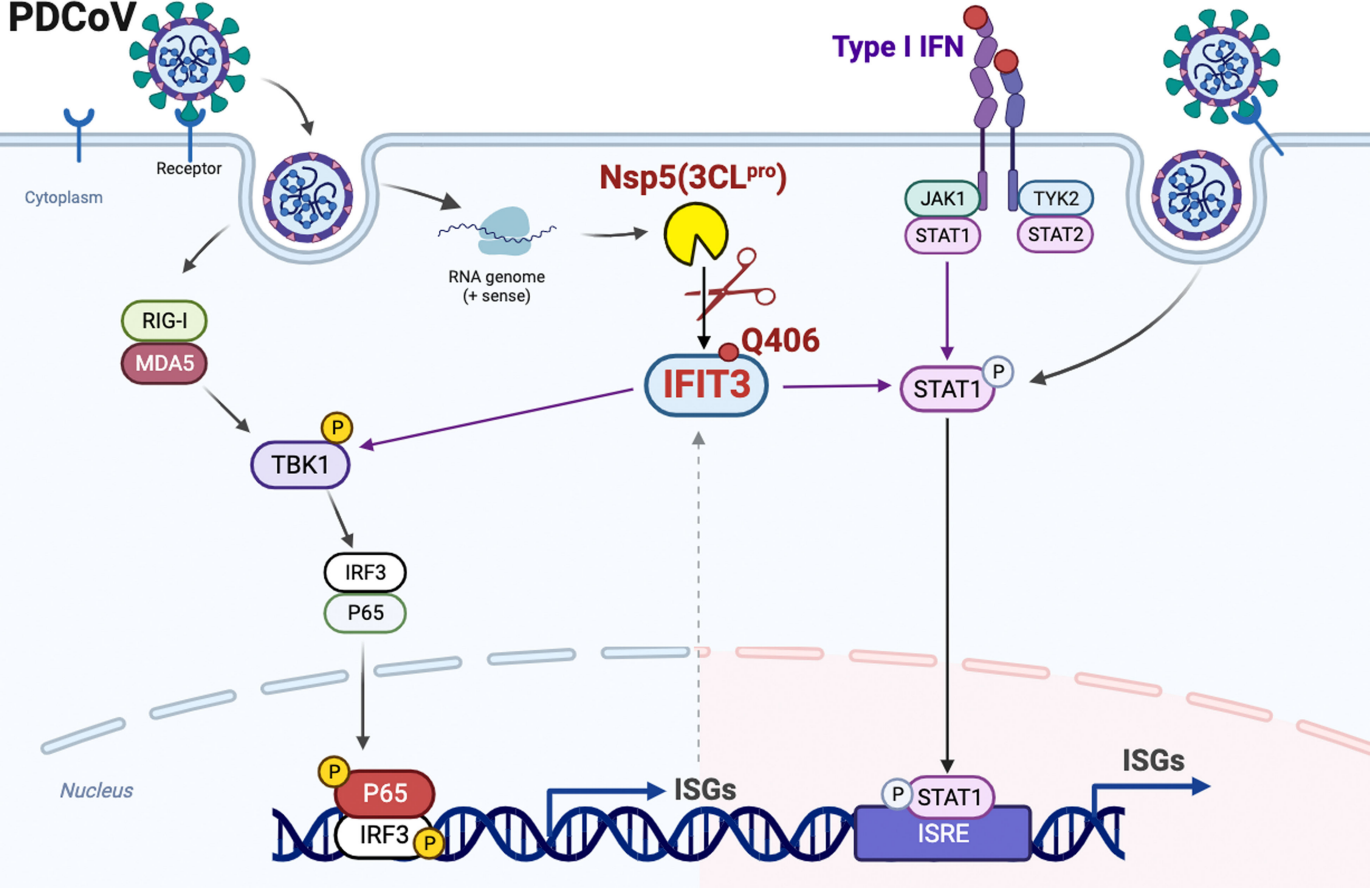
PDCoV nsp5 inhibits host innate immunity by directly cleaving IFIT3. Mechanistic diagram showing the antagonism of the IFIT3-mediated type I IFN signaling pathway by Nsp5 of PDCoV. The schematic was drawn using the Biorender website.

Here, we further validated the relationship between PDCoV replication and the host immune response (Fig. 8). Our findings demonstrated that pIFIT3 plays a protective role against PDCoV infection, and PDCoV nsp5 disrupts type I IFN signaling by cleaving pIFIT3, which requires the protease activity of PDCoV nsp5. The cleavage of endogenous IFIT3 in PDCoV-infected cells is consistent with the effects of nsp5 overexpression. In this study, we propose a novel mechanism by which PDCoV antagonizes the host antiviral response by cleaving IFIT3. To investigate whether IFIT3 cleavage activity is shared among the main proteases of different coronaviruses, we analyzed nsp5 proteins of porcine alphacoronaviruses (SADS-CoV), and three human coronaviruses (HCoVs): SARS-CoV-2, SARS-CoV, and MERS-CoV. IFIT3 could not be cleaved by nsp5 of SADS-CoV. Interestingly, IFIT3 was cleaved by the nsp5 proteins of all tested human coronaviruses (Fig. 7). Hence, determining whether cleavage of IFIT3 is unique to PDCoV nsp5 requires further evidence in other coronaviruses. Based on the identified residues of IFIT3 cleaved by PDCoV nsp5, establishing a cell line expressing IFIT3 proteins with mutated cleavage sites will be helpful in evaluating the functional significance of the potential cleavage of IFIT3 during PDCoV infection.

Among the excellent drug targets of coronaviruses are their main proteases (nsp5 proteins), which play a vital role in polyprotein processing to produce functional nonstructural proteins essential for viral replication and survival (43). For example, the main protease structure was used to screen the approved drug molecules to identify a candidate inhibitor of SARS-CoV-2 (44). It was reported that the endogenous POLDIP3 was similarly cleaved in the context of PDCoV infection in the IPEC-J2 cells, and reduction in endogenous POLDIP3 by cleavage was further corroborated in intestinal tissues from PDCoV-challenged SPF pigs *in vivo* (20). In addition, PEDV nsp5 can sustain virus infection by suppression pyroptosis via pyroptosis by cleaving gasdermin D (35). Regarding nsp5, an increasing number of papers are describing the method of substrate recognition for cysteine proteases in the chymotrypsin family, which primarily cleave peptides at the P2-P1-P1′ residues leucine-glutamine-alanine/serine (45, 46). For instance, the nsp5 proteins of feline infectious peritonitis, PDCoV, SARS-CoV, and SARS-CoV-2 target NF-κB essential modulator at a common cleavage site (Q231), resulting in inhibition of the type I IFN signaling pathway (34, 47, 48). SARS-CoV-2 nsp5 cleaved and inactivated the human tRNA methyltransferase TRMT1 at Q530 *in vitro* and *in vivo* (49). Our previous work demonstrated that the nsp5 protein of SADS-CoV cleaved DCP1A at a single site, Q343 (50). Here, our IFIT3 cleavage assay further demonstrated that the nsp5 proteins of porcine and human coronaviruses can target the same substrate [MKYQLQ (406) NGAAN]. Thus, determining the complete crystal structures of nsp5 and the cleaved peptide substrates of IFIT3 could provide more direct evidence and detailed information on the molecular interactions between the substrates and nsp5, facilitating the design of drugs targeting PDCoV nsp5.

In summary, we report that the nsp5 protein of PDCoV is a negative regulator of IFIT3-mediated type I IFN production. These findings provide a further explanation of the mechanism by which PDCoV nsp5 efficiently inhibits the host IFN response.

## MATERIALS AND METHODS

### Cells and viruses

HEK-293T (ATCC, CRL-11268), ST (ATCC, CRL-1746), and LLC-PK1 (ATCC, CL-101) cells were cultured at 37°C in a 5% CO_2_ atmosphere in Dulbecco’s modified Eagle’s medium (DMEM) (Invitrogen, USA) supplemented with 10% fetal bovine serum (Gibco, USA). The PDCoV strain, Sendai virus, and VSV-GFP were previously preserved in our laboratory. PDCoV was diluted in DMEM supplemented with 10 mg/mL trypsin and used to inoculate ST cells. After 2 h, the adsorbed PDCoV was discarded, and maintenance DMEM containing 10 mg/mL trypsin was added for incubation.

### Sample preparation and RNA-seq analysis

ST cells were seeded in 6-well plates (2 × 10^5^ cells/well) and infected with the PDCoV strain at an MOI of 1 for 24 h. Cells treated with the same volume of minimal essential medium for 24 h were considered mock infected. After infection, the cells were washed with 1× phosphate-buffered saline (PBS) (Solarbio, China) , and total RNA was extracted using the TRIzol reagent (Thermo Fisher Scientific, USA). For PDCoV-infected and mock-infected cells, three biological replicates were established per group. Total RNA was isolated and used for RNA-seq analysis. Transcriptome sequencing was conducted by OE Biotech Co., Ltd. (Shanghai, China). Bioinformatic analysis was performed using the OmicStudio tools.

### Plasmids and siRNAs

The gene-encoding PDCoV nsp5 was amplified from PDCoV strain CHN-HN- 2014 (GenBank accession number: KT336560) and was then cloned and inserted into the pCAGGS-flag-C vector. SADS-CoV nsp5 was previously preserved in our laboratory. SARS-CoV (GenBank accession number JX163928.1), SARS-CoV-2 (GenBank accession number OR587760.1), and MERS-CoV (GenBank accession number KU740200.1) genes were synthesized by Sangon Biotech (Shanghai) Co., Ltd. Full-length cDNA sequences of *Sus scrofa* (pig) IFIT3 (GenBank accession number NM_001204395.2), *Chlorocebus sabaeus* (monkey) IFIT3 (GenBank accession number XM_007963497.2), *Canis lupus familiaris* (dog) IFIT3 (GenBank accession number XM_038662411.1), *Homo sapiens* (human) IFIT3 (GenBank accession number NM_001031683.4), *Bos taurus* (cattle) IFIT3 (GenBank accession number NM_001075414.1) , *Sus scrofa* (pig) IFIT1 (GenBank accession number NM_001244363.1), *Sus scrofa* (pig) IFIT2 (GenBank accession number NM_001315658.1), *Sus scrofa* (pig) IFIT5 (GenBank accession number NM_001315572.1), *Sus scrofa* (pig) RSAD2 (GenBank accession number NM-213817.1), *Sus scrofa* (pig) USP18 (GenBank accession number NM_213826.1), *Sus scrofa* (pig) OAS1(GenBank accession number NM_214303.2), and *Sus scrofa* (pig) TRAF2 (GenBank accession number XM_005652719.2) were cloned and inserted into the pXJ40-HA vector. The Lipofectamine RNAiMAX Transfection Reagent and Lipofectamine 3000 Transfection Reagent were obtained from Thermo Fisher Scientific. siRNAs were synthesized by Tsingke Biotechnology.

### Dual-luciferase reporter activity assays

When HEK-293T cells in 12-well plates reached approximately 80% confluence**,** they were transfected with the firefly luciferase reporter plasmid (IFN-β-Luc) and Renilla luciferase reporter plasmid (pRL-TK) at a concentration ratio of 1:50. After 24 h, the cells were stimulated with SeV. The cells were lysed and analyzed with a luciferase reporter assay system (TransGen Biotech, China). Firefly luciferase activity was normalized to Renilla luciferase activity, and three biological replicates were established per group.

### RNA extraction and RT-qPCR

Total RNA was extracted by using TRIzol reagent following the manufacturer’s suggestions (Sangon Biotech, China). The extracted RNA was reverse transcribed into cDNA using PrimeScript IV 1st Strand cDNA Synthesis Mix (TaKaRa, Japan), and then mRNA expression levels were measured using RT-qPCR with SYBR qPCR Master Mix (Vazyme, China) in an ABI QuantStudio 3 real-time PCR system. Target gene expression was normalized to glyceraldehyde-3-phosphate dehydrogenase (GAPDH)expression. RT-qPCR primers are list in Table 1.

**TABLE 1:** Sequences of the primers and siRNAs

Primer	Forward (5′−3′)	Reverse (5′−3′)
h-IFN-β	TCTTTCCATGAGCTACAACTTGCT	GCAGTATTCAAGCCTCCCATTC
h-IFIT3	TCAGAAGTCTAGTCACTTGGGG	ACACCTTCGCCCTTTCATTTC
h-ISG54	CACCTCTGGACTGGCAATAGC	GTCAGGATTCAGCCGAATGG
h-ISG56	GCTTTCAAATCCCTTCCGCTAT	GCCTTGGCCCGTTCATAAT
h-IFIT5	GGCCAAAATAAAGACGCCCTT	GACCAGGCTTCGTACTTCTTC
h-RIG-I	TTCCCAGACCACAGGAATACC	GCAGGAGAACAAAGCCCAACT
h-MDA-5	TTCCGCTATCTCATCTCGTGC	ACTGTCCTCTGAATCTGCTCC
h-MAVs	GCCCATCAACTCAACCCGTG	TCCTCATTTCTGCTGCTCCC
h-IKBα	ACCTGGTGTCACTCCTGTTGA	CTGCTGCTGTATCCGGGTG
h-IRF3	AGAGGCTCGTGATGGTCAAG	AGGTCCACAGTATTCTCCAGG
h-NF-κB	TGGACCGCTTGGGTAACTCT	CCACCAGCAGCAGCAAACAT
h-GAPDH	TCTGCTCCTCCTGTTCGACAG	CCCAATACGACCAAATCCGTT
p-IFN-β	CATCCTCCAAATCGCTCTCC	ACATGCCAAATTGCTGCTCC
p-RIG-I	CTGGAGCTTGCTTTACCTGC	CCTTCCCCTTTCGTCCTTGT
p-MDA-5	GGAGTCAAAGCCCACCATCT	GCCACCGTGGTAGCGATAAG
p-ISG56	TCCGACACGCAGTCAAGTTT	TGTAGCAAAGCCCTGTCTGG
p-ISG54	GCACAGCAATCATGAGTGAGAC	CTGGCCCCTGCAGTCTTTTA
p-IRF9	AGCAGCAACAGCCCTGAGTC	CCCGTCGTAGATGAAGGTGAGC
p-RSAD2	AAAGACGTGTCCTGCTTGGT	CTTCCGCCCGTTTCTACAGT
p-CXCL10	ACTGTTCGCTGTACCTGCAT	GCTTCTCTCTGTGTTCGAGGA
PDCoV-N	AGCCACCCACCAAACCAACTA	CATCATCCCACTCCCAATCCT
p-GAPDH	AGCAACAGGGTGGTGGACCT	CTGGGATGGAAACTGGAAGT
PCA-C-PD-nsp5	CGAGCTCGCGGCCGCGGTAC CATGGCCGGCATCAAGATCC	CTTGTAATCACCTCCCTCGA G CTGCAGGCTGATGGGGGC
PD-NSP5-H41A	GCCGTGATCGGCAAGTTCAG GGGCGATCAGTG	AACTTGCCGATCACGGCCC TTGGGCAGTACACCACGT
PD-NSP5-C144A	TCCTGAACGGCGCCGCCG GCAGCGTGGGGTACACA	GGCGGCGCCGTTCAGGAA GCTGGCGTAGATCA
p-IFIT3-Q384A	AGCCCAAGCCTCTTTAGA AAATTTGTCCATAAACACG	CTAAAGAGGCTTGGGCTG CAGTGTCTTCAGAC
p-IFIT3-Q406A	CCAACTAGCCAATGGAGC TGCAAATCAGCTTC	CTCCATTGGCTAGTTGGTA TTTCATCTTTTCCTTCTCA
p-IFIT3-Q436A	ATGGAGACCTGCTGGCCG CAGCCGCATGCTATGAGA	GGCCAGCAGGTCTCCATT CATCTTGTGAATTA
pXJ40-human-IFIT3	CCCGATTACGCCTCCGGAT CC AT GAGTGAGGTCACCAAGAATTCC	GCTTTAATAAGATCTGGTAC CTCA GTTCAGTTGCTCTGAGTTAGAGAG
pXJ40-bovine-IFIT3	CCCGATTACGCCTCCGGATCCAT GAGTGAGGACAAGAATTCTCTGG	GCTTTAATAAGATCTGGTACC TCAGGGGTCGGGAAGCTC
pXJ40-dog-IFIT3	CCCGATTACGCCTCCGGATCCATG AGTGAGGTCAACAAGAATTCTCT	GCTTTAATAAGATCTGGTACCT CAGGTCTCAGGAGCTCTGAGAA
pXJ40-monkey-IFIT3	CCCGATTACGCCTCCGGATCCAT GAGTGAGGTCACCAAGAATTCC	GCTTTAATAAGATCTGGTACCT TAC TCTGAGTTAGAGACGAGCTCTCTAG
pXJ40-porcine-IFIT3	CCCGATTACGCCTCCGGATCCATGA GTGAGGTCAACAAGAATTCTCT	GCTTTAATAAGATCTGGTACCTCAG CCACTATTCCAGGCGC
si-human-IFIT3	GAUGUACCAUCUGGAUAAU	AUUAUCCAGAUGGUACAUC
si-porcine-IFIT3	GCAGGAGAAUCUGAAGCUATT	UAGCUUCAGAUUCUCCUGCTT

### Immunofluorescence assay

ST cells were transfected with the pXJ40-HA-pIFIT3, pCAGGS-flag-PDCoV-nsp5, or pCAGGS-flag-PDCoV-nsp13 expression plasmid. After 30 h, the cells were fixed with 4% paraformaldehyde for 15 min and then permeabilized with 0.1% Triton X-100 (Sigma-Aldrich, USA) for 15 min at room temperature. After three washes with PBS, the cells were blocked with PBS containing 3% bovine serum albumin (Solarbio, China) for 1 h and were then incubated separately with a rabbit anti-IFIT3 polyclonal antibody (Proteintech, China; 1:200) and a mouse anti-Flag tag antibody (Bioss, China; 1:200) for 2 h at room temperature or overnight at 4°C. Then, the cells were incubated with anti-mouse IgG (H + L), F(ab′)_2_ fragment (Alexa Fluor 488 Conjugate) (Cell Signaling Technology, USA) and anti-rabbit IgG (H + L), and F (ab′)_2_ fragment (Alexa Fluor 594 Conjugate) (Cell Signaling Technology, USA) for 1 h prior to incubation with 4′, 6-diamidino-2-phenylindole-dihydrochloride (Beyotime, China) for 10 min at room temperature. After rinsing in PBS three times, the images were acquired by direct forwarding confocal fluorescence microscopy.

### Western blot analysis and Co-IP

HEK-293T, ST, and LLC-PK1 cells were cultured in 6-well plates or 60 mm dishes and harvested with RIPA lysis buffer (Beyotime, China) supplemented with a protease inhibitor cocktail (Beyotime, China). Then, the cells were lysed by ultrasonication or incubation on ice for 15 min. The cell lysates were centrifuged at 12,000 × *g* for 10 min before the supernatants were either subjected to immunoprecipitation (IP) or directly denatured at 100°C for 10 min. Proteins were separated using 10% SDS-PAGE gel and transferred to a PVDF (Millipore, USA) membrane. For immunoblotting, the PVDF membrane was blocked in 5% skim milk (BD, USA) at room temperature for 1 h and was then washed with PBST three times. The membrane was incubated with primary antibodies for 2 h at room temperature or overnight at 4°C. After rinsing in PBST three times, the membrane was incubated with a secondary antibody at room temperature for 1 h. The membrane was rinsed to remove PBST and was then incubated with chemiluminescence reagent to detect the target protein. Overexpression of SADS-CoV, SARS-CoV, SARS-CoV-2, MERS-CoV, and PDCoV nsp5, and the PDCoV nsp5 mutants was evaluated using an anti-Flag antibody (Bioss, China). An anti-HA antibody (Proteintech, China) was used to analyze the expression of human, porcine, bovine, dog, and monkey IFIT3. An anti-GAPDH monoclonal antibody (Proteintech, China) , anti-STAT1 polyclonal antibody (Proteintech, China), anti-TBK1 antibody (Proteintech, China), anti-IRF3 antibody (Cell Signaling Technology, USA), and anti-p65 antibody (Cell Signaling Technology, USA) were utilized to detect each respective endogenous protein. An anti-phospho-STAT1 antibody (Cell Signaling Technology, USA), anti-phospho-TBK1 antibody (Affinity, China), anti-phospho-IRF3 antibody (Cell Signaling Technology, USA), and anti-phospho-p65 antibody (Cell Signaling Technology, USA) were utilized to detect the phosphorylated form of each respective endogenous protein.

ProteinIso Protein A/G Resin beads (TransGen Biotech, China) were pretreated with lysis buffer. As described above, the supernatant was added to ProteinIso Protein A/G Resin beads and incubated on a shaker at 4°C for 1 h. Then, an anti-Flag or anti-IFIT3 antibody was added to the pretreated supernatant. After incubation for 3 h at room temperature, the lysis buffer was washed away by three rounds of centrifugation at 1,000 × *g* for 5 min each. Then, the protein samples were prepared for Western blot analysis.

### Flow cytometry

HEK-293T cells seeded in 12-well plates (2 × 10^5^ cells/well) were transfected with the PDCoV nsp5 and mutant plasmids. After 24 h, the cells were stimulated with SeV for 12 h, a recombinant VSV-GFP was added to the cells, and the IFN bioassay was performed as described previously. The percentage of GFP-positive cells in the IFN bioassay was calculated with a Beckman Coulter DxFLEX flow cytometry system, and the data were analyzed by FlowJo v10.

### Sequence alignment

Amino acid sequences of IFIT3 were obtained, and the DNAMAN8 program was used for sequence alignment with the Lasergene sequence analysis software package (MegAlign) using Clustal W.

### Statistical analysis

All experiments in our study were conducted in triplicate. The results shown in the bar graphs were analyzed using GraphPad Prism 9 software, and the values are presented as the mean ± standard deviation of triplicates. *P* values <0.05 were considered to indicate statistically significant differences.

## SUPPLEMENTAL MATERIAL

Supplemental figures jvi.01682-23-s0001.docx Figures S1 and S2.

ASM does not own the copyrights to Supplemental Material that may be linked to, or accessed through, an article. The authors have granted ASM a non-exclusive, world-wide license to publish the Supplemental Material files. Please contact the corresponding author directly for reuse.
