# Supplementary material for: Porcine deltacoronavirus nsp5 antagonizes type I interferon signaling by cleaving IFIT3
Source: J Virol. 2024 Jan 30;98(2):e01682-23. doi: 10.1128/jvi.01682-23 (PMC10878044; doi:10.1128/jvi.01682-23)
Supplement: Supplemental figures — Figures S1 and S2. [file jvi.01682-23-s0001.docx]

**Supplemental Materials**

**Porcine Deltacoronavirus nsp5 Antagonizes Type I Interferon Signaling by Cleaving IFIT3**

Haixin Huang ^§a,b^, Xiaoxiao Lei ^§b^, Chenchen Zhao ^b^, Yan Qin ^b^, Yuying Li ^b^, Xinyu Zhang ^b^, Chengkai Li ^b^, Tian Lan ^b^, Baopeng Zhao ^b^, Wenchao Sun ^b#^, Huijun Lu ^c#^, Ningyi Jin ^a, c#^,

^a^ College of Veterinary Medicine, Northwest A&F University, Yangling, Shaanxi, China

^b^ Institute of Virology, Wenzhou University, Wenzhou, Zhejiang, China

^c^ Changchun Institute of Veterinary Medicine, Chinese Academy of Agricultural Sciences, Changchun, Jilin, China

Running title: PDCoV nsp5 cleaves IFIT3

#Corresponding author:

Prof. Ningyi Jin

Email addresses: [ningyik@126.com](mailto:ningyik@126.com)

Prof. Huijun Lu

Email addresses: [huijun_lu@126.com](mailto:huijun_lu@126.com)

Associate Prof. Wenchao Sun

Email addresses: [sunwenchao131@163.com](mailto:sunwenchao131@163.com)

^§^Haixin Huang and Xiaoxiao Lei contributed equally to this work. The author order was determined based on seniority.

**Supplementary Figures**

**
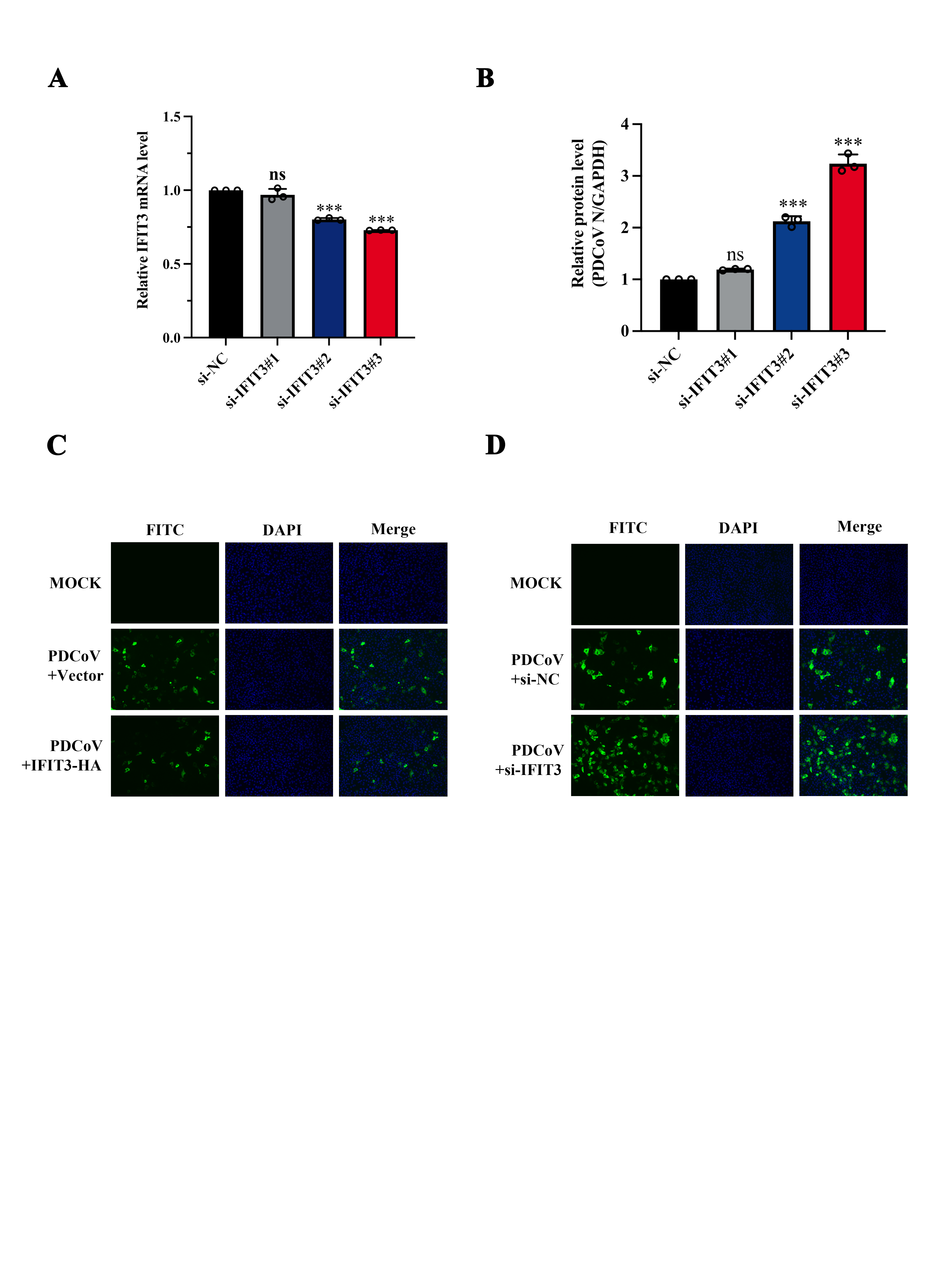
**

**Figure S1. Knockdown of pIFIT3 facilitates PDCoV replication**

(A) ST cells were transfected with negative control siRNA or one of three pIFIT3 siRNAs. After 36 h, the cells were lysed, and the IFIT3 mRNA level was measured by RT**-**qPCR. (B) Negative control siRNA and three pIFIT3 siRNAs were transfected into ST cells. After 24 h of transfection, the cells were infected with PDCoV (MOI=1) for 12 h, the cells were lysed, and the PDCoV N mRNA level was measured by RT**-**qPCR. (C) ST cells were transfected with pXJ40-HA-pIFIT3 or empty vector and were then infected with PDCoV at a MOI of 1. The cells were fixed and incubated with a mouse anti-PDCoV N monoclonal antibody (1:200) at 24 hpi. Immunofluorescence assays were used to further observe the intracellular propagation of PDCoV. (D) ST cells were transfected with negative control siRNA or si-IFIT3 and were then infected with PDCoV at a MOI of 1. The cells were fixed and incubated with a mouse anti-PDCoV N monoclonal antibody (1:200) at 24 hpi. Immunofluorescence assays were used to further visualize the intracellular propagation of PDCoV. All data are reported as the means ± SDs. For all experiments, **p* < 0.05, ***p* < 0.01, and ****p* < 0.001 were considered to indicate statistical significance. *ns*, nonsignificant difference.


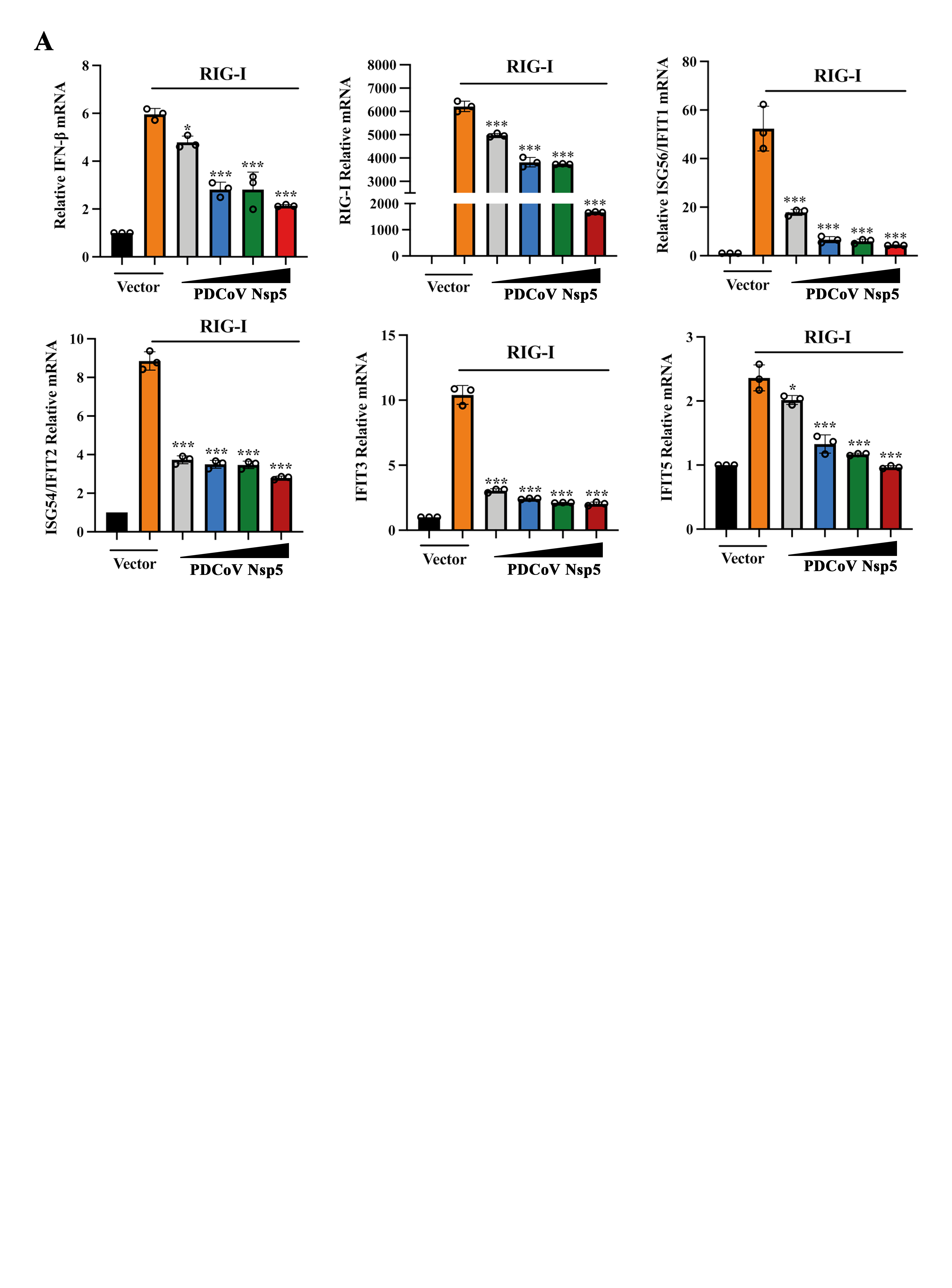


**Figure S2. PDCoV nsp5 inhibits RIG-I-mediated IFN-β and ISG production**

(A) HEK-293T cells were cotransfected with RIG-I and an empty vector or different amounts of the pCAGGS-flag-nsp5 plasmid. After 30 h, the cells were collected and the IFN-β, RIG-I, IFIT1, IFIT2, IFIT3 and IFIT5 mRNA levels were measured by RT**-**qPCR. All data are reported as the means ± SDs. For all experiments, **p* < 0.05, ***p* < 0.01, and ****p* < 0.001 were considered to indicate statistical significance. *ns*, nonsignificant difference.
